# From Disruption to Control: Insights from Focus Groups Exploring Nutrition and Chemosensory Changes During Menopause

**DOI:** 10.3390/nu17213411

**Published:** 2025-10-30

**Authors:** Sarah O’Donovan, Siobhan Monaghan, Aine Murphy, Paula Marie Conroy

**Affiliations:** Department of Sport, Exercise & Nutrition, Atlantic Technological University, ATU Galway City, Old Dublin Road, H91 T8NW Galway, Ireland; sarah.odonovan@atu.ie (S.O.);

**Keywords:** menopause, chemosensory changes, appetite, eating behaviour, nutrition, strategies for wellbeing

## Abstract

**Background:** Menopause is associated with metabolic, sensory, and psychosocial changes that may reshape eating behaviours and nutrition-related quality of life. This study explored how women experience nutrition and chemosensory changes during menopause and how these intersect with identity, control, and social practices. **Methods:** We conducted online focus groups (Microsoft Teams) with women living in Ireland (*n* = 40; mean age 58.3 years (±4.5 years)) between January and March 2025. Discussions followed a semi-structured guide focused on taste/smell, appetite, food choice, and coping. Sessions were recorded, transcribed, anonymised, and analysed following Braun and Clarke’s thematic analysis. **Results:** Four themes captured patterned meanings in the dataset: (1) Chemosensory Changes—reports of diminished taste, contrasted with heightened smell and selective intensification (sweetness), prompting compensatory behaviours (more salt/spice/strong coffee) and new aversions (e.g., cucumber, spicy dishes) alongside unexpected likes (e.g., dark chocolate); (2) Behavioural and Emotional Consequences—increased snacking, sugar/salt cravings, and perceived loss of satiety co-occurred with weight gain and altered body shape, undermining food pleasure and self-confidence; (3) Interacting Influences—affecting vasomotor symptoms, sleep disturbance, joint pain, and “brain fog” compounded dietary disruptions and social withdrawal (e.g., embarrassment about appetite, reduced desire to dine out); (4) Strategies for Wellbeing—women described medical approaches (HRT, prescribed medications) alongside food modifications and the importance of diagnosis, information, and peer/professional support. **Conclusions:** Menopause reshapes sensory perception and eating behaviour in complex, individualised ways that extend beyond biology to identity and social life. Nutrition care should integrate symptom management with person-centred strategies and improved access to evidence-based information, diagnosis, and support networks.

## 1. Introduction

Menopause is defined as the permanent cessation of menstruation, marking the end of a woman’s reproductive ability [1]. It typically occurs between 45 and 55 years of age, while early menopause is diagnosed before this range [2]. In Ireland, menopause is clinically diagnosed after 12 consecutive months without menstruation, with hot flushes often being one of the first symptoms experienced [3,4]. In 2024, the Health Service Executive (HSE) introduced a national Menopause Policy to support women experiencing menopausal symptoms and improve their quality of life [5]. The transition through menopause can be challenging, with symptoms ranging from vasomotor disturbances to cognitive complaints such as memory impairment in the early stages [6].

Common symptoms include hot flushes, insomnia, mood fluctuations, weight gain, and joint pain [3]. Hot flushes, among the most commonly reported symptoms, are linked to thermoregulatory dysfunction caused by fluctuating gonadal hormones, particularly oestrogen [7]. Dietary factors such as spicy foods, caffeine, and sugar may exacerbate vasomotor symptoms including hot flushes and night sweats [8,9]. Reviews also suggest that certain nutrients and phytoestrogen-containing foods can influence the severity of these symptoms [10,11,12].

Weight gain is also a well-documented consequence of menopause, with an increase in overall body fat, particularly in the abdominal region, contributing to elevated cardiovascular risk [13,14,15]. Altered dietary habits, such as increased intake of sugar and salt, have also been observed; however, limiting these components may help reduce symptom severity and associated health risks [16,17]. Dietary interventions, including those promoting higher consumption of vegetables and fresh foods, have been linked to improved mood, concentration, and memory [11,18]. Recent narrative reviews in Nutrients highlight the crucial role of diet in managing menopausal symptoms and overall wellbeing [19,20].

Mood changes and depression are prevalent and may be influenced by diet quality, with evidence linking high sugar consumption to impaired dopamine regulation and emotional dysregulation [21]. Conversely, phytoestrogen-rich diets and omega-3 polyunsaturated fatty acids have shown promise in improving vasomotor symptoms and mood during menopause [10,22]. Soy isoflavones, in particular, have been associated with positive effects on postmenopausal health [11], and vitamin E supplementation has also been explored as a non-hormonal intervention [23].

HRT remains the primary evidence-based treatment for managing menopausal symptoms and is available in various forms, including oral tablets, transdermal patches, gels, sprays, and vaginal rings [3,24]. HRT typically combines oestrogen, progesterone, and occasionally testosterone, and has been shown to significantly improve quality of life during the menopausal transition [25]. Intrauterine systems such as the Mirena coil are also utilised to prevent oestrogen-related endometrial hyperplasia while alleviating symptoms [26]. Herbal remedies, such as sage and evening primrose oil, are frequently used by women; however, scientific evidence supporting their efficacy remains limited [27].

Menopause is characterised by a decline in oestrogen and progesterone production, contributing to vasomotor, metabolic, and psychological symptoms that may influence diet and overall wellbeing [28,29]. Approximately 75% of women experience multiple symptoms during this transition [30]. Menopause can be divided into three stages: perimenopause, menopause, and postmenopause [31]. Perimenopause marks the transitional years before menopause and includes early and late stages, with fertility still possible though significantly reduced. Menopause is confirmed after 12 months of amenorrhea, usually occurring between ages 49 and 62 [32], after which women enter postmenopause [33]. These hormonal fluctuations can be difficult to distinguish from normal ageing processes, complicating symptom attribution [34].

Sensory function, particularly taste and smell, plays an essential role in dietary preferences, appetite regulation, and nutritional status [35,36]. Age-related sensory decline is associated with poor diet quality, while menopause-related hormonal changes may further contribute to dysgeusia, dry mouth, burning sensations, and altered taste perception [37]. Such changes can increase preference for high-salt foods, elevating the risk of hypertension and other cardiometabolic conditions. Furthermore, decreased oestrogen may influence dietary behaviours, potentially leading to increased fat and sugar consumption and subsequent weight gain [38].

Given the potential implications of chemosensory changes on diet quality, nutrient intake, and overall health, this study aimed to evaluate whether sensory decline, particularly in taste perception, occurs during and after menopause. It further explores the impact of these alterations on dietary behaviour and quality of life among menopausal women.

## 2. Materials and Methods

### 2.1. Study Design and Research Philosophy

This study makes use of pragmatic research philosophy [39], using real-life experiences, values, and beliefs to shape research and insight on sensory decline caused by menopause. Qualitative research was conducted via focus groups on sensory decline during and after menopause. This study design is based on a similar study conducted by Duffy, Iversen, and Hannaford (2011) that discussed symptoms, experiences, and management strategies of menopausal women [40]. Their study was completed through four focus groups of six women. The utilisation of focus groups allowed for rich, interactive discussions capturing multiple perspectives on the research topic, while also providing in-depth data in a time-efficient way. The focus group method provided an ideal setting for participants to reflect, compare, and build upon one another’s insights. The use of thematic analysis using the Braun and Clarke framework allowed for a subjectively immersed experience for the researcher, where this tool allowed for in-depth, explorative reporting and interpretation [41,42]. The use of a semi-structured focus group allowed for flexibility on how sensory decline impacted different women, investigating the relationship between menopause, sensory change, and nutrition practices.

### 2.2. Participants and Recruitment

Following ethical approval from the Atlantic Technological University Taught Programme Research Ethics Committee, aligning with the Declaration of Helsinki forty women were identified using convenience and snowball sampling methods. Snowball sampling was chosen as it is considered a standard technique in qualitative studies, especially when collecting data that may be difficult to obtain from various populations [43]. Convenience sampling is considered a time-efficient method, along with being convenient for researchers when gathering homogeneous populations [44]. Convenience and snowball sampling were employed to enrol midlife women undergoing menopause, due to the difficulties in approaching this demographic for conversations on sensitive health and sensory issues. Convenience sampling facilitated the inclusion of readily available and motivated participants, whereas snowball sampling expanded recruitment via personal and communal networks. These methodologies enabled prompt data acquisition but may have engendered bias by yielding a relatively homogeneous sample, as participants likely possessed analogous social origins, health literacy levels, or perspectives on nutrition and menopause. Such commonalities may have affected the diversity of opinions spoken and, as a result, the themes recognised. To mitigate this effect, recruitment commenced from several sources (e.g., social media, community organisations, and professional networks), and reflective analytical methodologies were upheld. Focus groups were conducted using Microsoft Teams, where email was used to provide documentation and invitations. A Microsoft Forms was also provided to the participants to select a date that suited them to partake in the study. On completion of the consent form, participants were allocated to a focus group and sent a meeting invite. Inclusion criteria stated women must be >45 years and experiencing one of the three stages of menopause, the participants were also eligible if they did not fall under the category of sensory impairment such as anosmia (loss of smell) or ageusia (loss of taste) due to cancer or other health conditions [45]. The participants were excluded if they were on medication that contributes to sensory impairment, if they were <45 years, or if consent was not provided. In total, forty-three women were invited to partake, and forty completed the focus groups; all participants were based in Ireland.

### 2.3. Procedure

The focus group guide, as outlined in Table 1, was designed from information that was used by the current research available, whilst also referring to the case study that was provided in the information form. The case study (Appendix A) was created using real-life experiences, by communicating with an individual currently going through menopause as well as consulting with past papers on various symptoms that may arise, to ensure all participants may relate to a symptom from the case study. The questions created were targeted towards chemosensory changes, particularly changes in taste, to assess if there was a correlation with menopause and the decline of the senses. Open-ended questions were used to enable the researchers to capture multiple perspectives. Phrases such as “Anything in particular?” and “Is it to get the taste?” were used. These clarification probes are used in order to gain clarity and an understanding of what has been stated by a participant [46], as well as to possibly prompt other participants to recall similar situations. This also enabled the researchers to capture multiple perspectives of what was being described in the focus groups, while promoting further discussion and interaction among the participants. The focus groups were recorded, transcribed, and anonymised by assigning participants a numerical code. Data was collected between January and March 2025, and the focus groups lasted between 33:38 and 1:00:15 min (44.13 ± 9.06 min).

### 2.4. Data Analysis

Data obtained from the focus group was analysed by using the Braun and Clarke thematic analysis (TA) method [42]. The purpose of TA in qualitative data collection is to ensure the trustworthiness of qualitative data in a clear manner, while identifying, organising, analysing, describing, and reporting certain themes and sub-themes that may arise within the data [47]. This typically reflects personal experiences and various situations which allow for creating an understanding of different topics from an external perspective [48], such as taste changes during the menopausal transition.

This form of data analysis followed a six-step process. Step 1 consisted of becoming familiar with the data; this was completed by re-watching the focus group recordings to clean the transcripts. Possible codes were noted throughout the focus groups as well as while combing through the data. Step 2 entailed coding the data; this was done whilst taking notes of possible themes within the data and compiling them to create an overall coding framework. In the third step, the initial codes that formed were then analysed, and the generation of initial themes was performed. Once that was completed, step 4 allowed for the overall development and review of themes. Identified themes were checked against coded data to determine if the themes were legitimate. This step allowed some previously identified themes to be split from the assigned theme, combined with another theme, or discarded due to a low pattern arising. In step 5, the themes were further refined with clear definitions and names for each dataset that emerged. Finally, step 6 was where all analysed data is extracted and contextualised to create a scientific paper [42]. Quotes were then used to support the themes that were chosen to enhance the results found.

### 2.5. Poistionality and Trustworthiness

The project team consisted of a registered associate nutritionist (ANutr), two undergraduate Public Health Nutrition students, and a registered nutritionist (RNutr), all based in Ireland. Their involvement provided multiple benefits, including fostering rapport and creating a comfortable, judgement-free environment where participants felt comfortable sharing their experiences, as well as offering fresh perspectives on the research questions and analysis. The age and life-stage differences between the student interviewers and the participants may have influenced the group dynamics. To mitigate these potential limitations, the students underwent training in qualitative interviewing, maintained an empathetic stance throughout discussions, and engaged in regular debriefings with the senior researchers to reflect critically on their positionality and influence on the data.

Proven strategies must be adhered to in order to produce a high standard of data analysed by TA [49], where the researcher is immersed in the dataset. Throughout the analysis, sub-themes and themes were assessed, edited, combined, or deleted to identify the higher-order themes. The use of TA is a flexible and accessible method for analysing qualitative data, particularly due to its subjectivity. This method allows for reliable coding of transcripts and data, leading to accuracy of results [50].

The primary research question was formulated by the research team as an element of a broader study on menopause and the changes women undergo. This area of applied research is significantly underexplored, especially regarding sensory alterations. The principal author possesses practical expertise and a keen interest in nutritional practices and sensory alterations in the ageing population, and comprehends relevant challenges encountered by practitioners in this field. Throughout the formulation of the interview guide and the ensuing data collection, the lead author sought to distinguish her personal experiences and beliefs from those of the participants, maintaining objectivity.

The research team’s positionality is perhaps a significant strength of the study. Positionality is associated with reflexivity and recognises that a researcher’s social and personal identities, experiences, and views interact, thus influencing their research. The acknowledgement of this is significant as it fosters research creativity [51].

## 3. Results

Forty women residing in Ireland participated in the study (*n* = 40). The average age of participants was 58.3 years (SD = 4.5). All participants classified themselves as White Caucasian. Thirty percent of the ladies, totalling twelve, were presently on hormone replacement treatment (HRT). Concerning menopausal state, 27 participants (67.5%) were postmenopausal, 12 (30%) were menopausal, and 1 (2.5%) was perimenopausal (Table 2).

Four themes were identified from the thematic analysis of the dataset, each with sub-themes illustrating patterns in experiences and perspectives: 1. Chemosensory Changes, 2. Behavioural and Emotional Consequences, 3. Interacting Influences, and 4. Strategies for Wellbeing. Each theme is supported by exemplar participant quotes in Table 3.

### 3.1. Chemosensory Changes

Participants’ accounts revealed that menopause was experienced not only as a biological transition but also as a sensory re-orientation, one that reshaped their relationships with food, appetite, and identity. These chemosensory changes were multifaceted, sometimes manifesting as loss, sometimes as intensification, and carried important emotional and social consequences. Four sub-themes captured this complexity: diminished sensory perception, heightened sensory perception, appetite and eating habits, and food preferences.

#### 3.1.1. Diminished Sensory Perception

For some women, menopause was marked by a fading of sensory pleasure. Food was described as “bland” or like “cardboard” (A001), and previously enjoyable meals no longer provided satisfaction (see Table 3, Quote 1.1.2.). These losses were often narrated as disappointments, symbolising not only a change in taste, but also a broader sense of diminished vitality. The decline in sensory richness disrupted routines of comfort and reward, undermining food’s role as a source of pleasure and social enjoyment:

*“I’m literally eating for the sake of eating because everything is just so bland. It doesn’t taste of anything.”*

(B004)

Interestingly, the persistence of exaggerated sweetness highlighted that chemosensory changes were uneven, producing a confusing landscape where the familiar no longer felt stable (see Table 3, Quote 1.13).

#### 3.1.2. Heightened Sensory Perception

In contrast, other participants experienced heightened sensitivity, particularly to smell. Odours became intrusive, with one woman remarking that she could detect onions “coming down the road” (B005). Such accounts conveyed a loss of control over one’s bodily boundaries, where everyday environments became sources of discomfort or even illness (e.g., migraines). These sensory amplifications carried practical consequences such as discarding food perceived as spoiled before its use-by date (see Table 3, Quote 1.2.4) but also altered emotional relationships with spaces once associated with comfort, like kitchens or living rooms. Changes in taste sensitivity produced similar tensions. While some mourned their inability to tolerate spicy curries (see Table 3, Quote 1.2.7), others discovered intensified enjoyment of chocolate and cheese (see Table 3, Quote 1.2.1). These contradictory accounts underscored menopause as a destabilising period where sensory reliability and, by extension, bodily identity were called into question.

#### 3.1.3. Appetite and Eating Habits

Increased snacking was commonly reported. Women described grazing “*all evening*” (see Table 3, Quote 1.3.1) or becoming “*constant grazers*” and snackers (see Table 3, Quotes 1.3.2 and 1.3.3). Appetite changes were described as destabilising, overwhelming, and difficult to manage socially. For some, hunger felt insatiable despite attempts to regulate it through “healthy” eating:

*“My appetite… I couldn’t quench it. I was hungry all the time, even eating loads of protein… I wasn’t satisfied”*.

(P864)

Some participants later noted being able to go five hours or more without eating and not being hungry, which highlighted the unpredictability of these shifts. For others, persistent hunger was experienced as embarrassing:

*“Still, I’m so hungry, and if I’m eating with people, I’m embarrassed… so I might go home and have something else. But, like, my appetite is just ridiculous”.*

(P110)

This tension between bodily need and social acceptability demonstrated how menopause complicated women’s identities as eaters, creating shame around behaviours that challenged norms of restraint.

A sense of loss of control was also evident:

*“I eat a lot more. I can’t stop eating and I could eat what somebody else hasn’t finished on their plate”.*

(P110)

And this participant also described out-eating her partner (see Table 3, Quote 1.3.7). These accounts illustrated how shifts in appetite disrupted gendered expectations around food, leaving women negotiating feelings of excess and frustration. Hunger was not merely physiological but intertwined with identity, social comparison, and expectations of moderation.

Cravings for sugary foods were frequent and often linked directly to menopause. The women described intense desires for sweet foods:

*“I love sweets. And definitely since the menopause I would be sweet mad. Sweet, sweet mad”*.

(P101)

Others described a new preference for biscuits and cakes, stating that they had increased their consumption and purchasing of these products. Others noted a shift in preference from savoury to sweet foods (see Table 3, Quote 1.3.10). Chocolate cravings were common (see Table 3, Quote 1.3.11), often forming part of evening rituals that brought both feelings of comfort and frustration.

Many participants also described an intensified preference for salt (see Table 3, Quotes 1.3.12 and 1.3.15). This reflected a search for stronger flavours and a perceived dulling of taste. Aromat was a recurrent seasoning choice. Some cravings were extreme:

*“Yeah, love salt. I’d put salt on ice cream”.*

(P252)

Others described repeatedly salting meals (see Table 3, Quote 1.3.14). These shifts were persistent and often publicly visible, provoking self-consciousness. Women contrasted current habits with previous habits:

*“At one time I never used salt in cooking… but now I would find that I’d need salt or need to flavour things a bit more”.*

(P614)

Salt was thus framed as essential to restoring flavour, reflecting both physiological change and social negotiation.

#### 3.1.4. Food Preferences

Many participants described a shift towards seeking stronger, bolder flavours during menopause, often portraying this as both a response to diminished taste and a new sense of experimentation. For example, women started to introduce chilli to enhance flavour:

*“I want some chilli on everything”.*

(C002)

Increasing flavour intensity in individual food and meals, for example, by adding “that extra spoon of coffee” to heighten taste, was a common topic of discussion (see Table 3, Quote 1.4.3). For some, foods once disliked became appealing:

*“Before I used to hate anything with chilli in it. Forget it. Now, just give it to me. I’m fine”.*

(B004)

Whilst for others, the need for stronger flavours became a key factor in food preference. Such changes represented both sensory adaptation and identity renegotiation through food experimentation.

Cravings for sugar and salt reinforced struggles for control. Participants described becoming “*sweet mad*” (P101), ritualising chocolate consumption (B001), or salting ice cream (P252). These narratives conveyed humour and discomfort, as altered appetites disrupted long-standing eating habits.

Alongside intensified cravings, the women reported new aversions. Previously enjoyed foods became intolerable:

*“And I used to love cucumber… I just find it disgusting… everything about it, just the smell, the taste, it just makes me heave”.*

(C002)

Spices also triggered aversion (see Table 3, Quote 1.4.7). Some women rejected red meat they had once enjoyed. These aversions carried emotional weight, reshaping daily food practices and identities.

*“I used to eat a steak every time, I loved it. Just couldn’t look at it now, I have no interest in the world”*.

(P651)

Conversely, some participants described new likes, often with surprise:

*“I actually now eat gherkins. I never liked gherkins. Well, I do eat gherkins, but these are the vinegary taste.”*

(P864)

Another surprising food preference shift was the change in preference for dark over milk chocolate, especially when adding novel ingredients to chocolate, for example, frozen ginger. Such reflections framed menopause as a period of adaptation and renewal, as women incorporated new tastes and redefined their culinary identities (see Table 3, Quote 1.2.12).

### 3.2. Behavioural and Emotional Consequences

Alongside chemosensory changes, participants described menopause as a time of behavioural and emotional disruption, particularly in relation to weight, body image, food enjoyment, and the availability of social support. These experiences were often narrated in terms of frustration, loss of control, and isolation, reflecting how menopause reshaped everyday practices and self-perceptions. Three interconnected themes captured these dynamics: weight gain and body image, functionality versus pleasure, and support networks. Two key sub-themes emerged: weight gain and body image, highlighting the distress and frustration linked to physical changes and altered eating patterns, and support networks, underscoring the significance of information, peer connections, and professional guidance.

#### 3.2.1. Weight Gain and Body Image

Weight gain emerged as one of the most consistent and distressing narratives. Many participants spoke of sudden, uncontrollable increases, often framed as both bewildering and disempowering. One woman explained: *“The weight has heaped on, the minute I hit 50, I felt like I gained about an extra stone and a half like in a week”* (C003). For others, the change was framed as cumulative and relentless (Table 3, Quote 2.1.3). Weight gain was widely attributed to menopause, with women expressing frustration at how easily weight accumulated despite efforts to manage it: “You definitely put on weight way easier, I watched what I ate” (P922). For some, this shift disrupted ongoing weight management journeys, leaving them feeling demoralised and socially withdrawn. As C004 explained:

*“My weight loss journey was so, so different to what the girls are feeling now because I put on so much weight… I didn’t want to go anywhere”.*

(C004)

Body shape changes were also significant. Even women who had not experienced major weight increases described noticeable shifts in the body parts that accumulated weight (see Table 3, Quotes 2.1.7, 2.1.8, and 2.1.9). For others, the experience was tied to appetite changes, with one participant reflecting:

*“I’ve gone up a stone and a half… our body will actually make you eat more because it wants more fat”.*

(P864)

Cravings, particularly for sugar, were often blamed. P311 noted, “The craving was always there for the sugar, the sugary stuff you know, like the chocolate,” while P110 admitted, “I eat a lot more. I can’t stop eating and I could eat what somebody else hasn’t finished on their plate” (P110). Together, these accounts highlight how weight gain was experienced not simply as a bodily outcome but as a challenge to self-control, identity, and social participation.

#### 3.2.2. Support Networks

Women also reflected on the role of support, or its absence, in shaping their experiences. For some, family history created expectations about the timing and severity of menopause. As P791 explained:

*“Mammy had gone through it young and it looked like there was a more chance of me going through it earlier as well”.*

(P791)

Similarly, A004 linked her own journey to her mother’s, noting: “Family history… you find if people’s mother goes through the menopause early” (A004). In contrast, others expressed the loneliness of lacking family to confide in: “Some of us may not have had anyone to talk to” (P651). Outside of family, participants emphasised the value of information and peer support:

*“If I had my time over I would definitely arm myself with more information and there’s support groups… chatting about it tonight, if those people can go on to different forums and you know get help through groups… definitely would make a difference”.*

(P252)

Yet for many, menopause remained shrouded in silence, with P651 noting: “Apart in my doctor’s office I’ve never spoken openly about the subject to anyone” (P651). In response to this lack of dialogue, some women sought knowledge independently. P864 explained: “I’ve done a lot of research into the menopause and I’m just about to do a course… all those can be a sign of low oestrogen” (P864). For those most affected, professional help became necessary, with C005 sharing: “I went to a menopause specialist… because I really was struggling” (C005). These accounts show that while family history and peer support could provide grounding, many women navigated menopause in isolation. Where resources and networks were available, they offered validation and practical coping strategies; where absent, women’s experiences were marked by confusion, silence, and struggle.

Across these themes, the behavioural and emotional consequences of menopause were experienced as deeply embodied and socially situated. Weight gain and body shape changes unsettled women’s identities and sense of control, while the loss of food pleasure represented a broader diminishment of everyday enjoyment. Support networks or their absence played a critical role in mediating these experiences, shaping whether women felt isolated or empowered to adapt. Ultimately, participants’ accounts revealed menopause not as a purely biological process but as one that reconfigured relationships with food, body, and community.

### 3.3. Interacting Influences

A combination of physical, emotional, and social challenges was experienced with menopause, with each influencing the others in complex ways. This theme was broken down into three sub-themes: physical symptoms, emotional wellbeing, and social wellbeing.

#### 3.3.1. Physical Symptoms

Participants reported a wide range of physical changes during their menopause experience, most commonly sleep disturbances, joint pain and stiffness, hot flushes and sweats, and discomfort after eating. These symptoms often disrupted daily routines, contributed to fatigue, and reduced quality of life.

Hot flushes and sweats, commonly associated with menopause, were one of the main issues the women faced during their menopause journey. At any point during their day, no matter what they were doing, they could suddenly break out in a sweat, and their clothes would be sticking to them. The experience was described by one woman “like a volcano gone off inside” (P126). These instances of sudden hot flushes and sweats which often resulted in reddening faces and damp clothing left the women feeling embarrassed and affected their confidence on a daily basis (see Table 3, Quote 3.1.6.). Nighttime sweats also contributed to a lack of sleep alongside other reported issues such as leg cramps. These symptoms at night caused the participants to lose hours of sleep on a regular basis, with some women reporting getting on average as little as two hours of sleep a night:

*“Do you know the way the way medical experts will tell you, you should be getting seven and eight hours sleep a night. Well, that’s next to impossible for someone going through menopause. I find you might get two. You’re awake.”*

(P413)

This broken sleep pattern led participants to seek sleeping aids, turning to both holistic supplements and prescribed medication, in an effort to have a full night’s rest and wake up feeling refreshed. Along with fatigue, a common experience each morning was regular joint stiffness, particularly in the hips, and the feeling of having become misaligned during the night (Table 3, Quote 3.1.3.). The women suffered a heavy toll from these regularly occurring symptoms which left them fatigued and drained of energy to go about their daily routines.

*“It’s awful to think that you have to rely on a tablet or medication to get sleep. There has to be something else out there. For women to be able to relax and get rid of these hot flushes and sweats, there has to be something else besides medication”.*

(P311)

During menopause, women noticed a notable shift in their relationship to food, with previously well-tolerated staples such as bananas now provoking heartburn, regurgitation, and stomach discomfort, highlighting the embodied, everyday disruptions of this life stage. Because eating is a daily act, these changes were experienced constantly, reinforcing menopause as a lived, ongoing presence rather than an occasional episode. Foods that once were considered enjoyable and brought a sense of joy and comfort such as Banoffee Pie were removed from their diets in an effort to lessen any discomfort from eating (Table 3, Quote 3.1.7.). Such experiences often forced women to exercise greater vigilance and caution with food choices, reshaping their everyday practices.

#### 3.3.2. Emotional Wellbeing

Emotional challenges were described as equally disruptive, including a lack of motivation, loss of identity, and difficulties with concentration or “brain fog”. These experiences were described as undermining confidence and creating feelings of frustration or diminished self-worth.

Reflections on a lack of motivation around both physical activity and food highlighted how menopause extends beyond physical symptoms to affect emotional engagement with daily life and routines. The reported loss of flavour or altered sensory perception of foods diminished their enjoyment of eating, stripping away the pleasure once associated with meals and leading to disinterest in food choices and preparation. Eating, which often carries social, cultural, and emotional significance, became less about enjoyment and more about necessity, leaving participants with a sense of detachment from practices that once enriched their lives.

*“I probably don’t enjoy food now, whereas I did before.”*

(B004)

For others, their lack of motivation to “get up and go” was more closely linked to their loss of identity during their menopause journey. Participants experienced a knock to their confidence by the changes their bodies were undergoing and wished they could revert to their bodies before menopause. Weight gain and tiredness in particular were noted as the main culprits for diminished confidence and self-worth, leaving participants feeling disconnected from their ‘old self’ and reducing their interactions in public (Table 3, Quote 3.2.5.). Further compounding the participants’ changing self-perception was the challenge of experiencing “brain fog”. This experience was highlighted as regularly inducing feelings of confusion, embarrassment, and frustration during their daily routines when the “brain fog” set in and caused the women to forget the names of things, people, and places (Table 3, Quote 3.2.6.).

#### 3.3.3. Social Wellbeing

The women’s social lives were also affected during menopause. Participants spoke of embarrassment over changes in appetite, which led to some avoiding dining out, while others described a reduced desire to engage in social activities altogether. Embarrassment over changes to appetite was highlighted in relation to increased hunger and the perception that saying “I’m still hungry” would not be socially acceptable or would draw unwanted attention upon the individual. In these instances, participants often reported going home and eating additional food in private. Changes to taste and enjoyment of food, as well as discomfort with body image and the impact on the participants’ self-confidence, left women feeling a lack of desire to eat out in public. An inability to taste and enjoy the food they were paying for meant some women considered eating out a waste of time and money:

*“No, no, no, ‘cause. You just don’t taste it. But I don’t taste it, you know? So there’s no point. There really isn’t any point, you know.”*

(B004)

These social shifts compounded feelings of isolation and contributed to a sense of disruption in lifestyle. Together, these sub-themes illustrate how physical symptoms, emotional wellbeing, and social contexts intersect, creating a cycle of challenges that extends beyond biological changes alone.

### 3.4. Strategies for Wellbeing

Despite these challenges, participants identified a range of strategies for managing and adapting to menopause. These strategies were both medical and lifestyle-based, reflecting efforts to regain a sense of control and balance. This theme was broken down into three sub-themes: treatment—medical and holistic; food modification and conscious eating; and diagnosing.

#### 3.4.1. Treatment—Medical and Holistic

Women described using a combination of hormone replacement therapy (HRT), medication, supplements such as magnesium, and holistic approaches including sage and magnets. HRT was a passionate topic of conversation across all focus groups, with women describing it as life-changing once they started HRT and how their symptoms, such as mood swings, hot flushes, and ringing in the ears, were alleviated (Table 3, Quote 4.1.2.). Participants reported feeling relief after starting HRT, and that they felt like they had regained control and balance over their mental and emotional states (Table 3, Quote 4.1.3.). Holistic approaches to treatment of symptoms were also discussed, with the use of sage and magnets to alleviate hot flushes and sweats mentioned. Other holistic approaches included the use of evening primrose oil to reduce the severity of hot flushes and night sweats. These holistic therapy approaches were generally reported as beneficial for reducing the frequency and intensity of hot flushes and sweats both during the day and at night.

Another treatment approach that was brought up across the focus groups was the use of magnesium to reduce instances of leg cramps. Magnesium was positively discussed by participants in alleviating leg cramps and joint stiffness, thereby allowing women to enjoy a better night’s rest. However, it was noted that the use of magnesium to treat these menopause symptoms should be under the guidance of a healthcare professional such as a GP or a licensed medical herbalist:

*“You have to be careful that you need to be led by a herbalist or something, because if it’s not that problem, you’ll create a new problem by taking it (magnesium)”*

(P252)

Prescription medications were also cited as part of individual management strategies, with choices often guided by personal preferences and perceived effectiveness. Anxiety was often identified as a side-effect of the physical symptoms, in particular hot flushes, as well as the impact on emotional and social wellbeing. Participants who experienced this often sought medical help to relieve their anxiety and were prescribed medication that was appropriate to support their wellbeing. Other participants, who had been using the Mirena coil contraceptive, were recommended by their GPs to continue its use into menopause due to the coil releasing levonorgestrel, which can act as a form of progesterone therapy, replacing some of what is lost during menopause (Table 3, Quote 4.1.5.).

#### 3.4.2. Food Modification and Conscious Eating

Dietary changes were identified as an important strategy, with some participants describing deliberate improvements in food quality, while others placed emphasis on conscious consumption, including smaller portion sizes and mindful eating practices. These adjustments were often linked to managing appetite, digestion, and energy levels.

It should be noted that while some participants described a strong liking for chilli and other spicy foods (see Section 3.1.4), others reported avoiding such flavours due to intolerance. This variation reflects individual differences in taste preferences and sensitivity among participants. Spice and spicy foods were regularly mentioned as intolerable during menopause, resulting in many women opting to avoid spice in their diets to alleviate the symptoms associated with its consumption. Women mentioned having to opt for milder dishes like Spaghetti Bolognese instead of a curry (Table 3, Quote 4.2.1.). Intentional improvements to diet quality by opting for healthier foods to improve their overall health, as well as support their menopausal symptoms, were a point of discussion for the participants. Women described seeking low-sugar, low-fat foods and food alternatives when shopping or eating out, as well as opting for “fresher and healthier” food. It was understood that eating healthier could be the difference between feeling better or worse, and that food choices impacted the severity and frequency of some symptoms:

*“There’s a lot of information out there now, it seems that definitely diet is a huge part of how smooth or how rough it’ll be (menopause).”*

(P252)

Conscious eating was identified as a useful approach to support overall wellbeing in menopause. By eating mindfully and consuming smaller portions, women reported being able to better manage their weight, increased their enjoyment of food by taking the time to “taste it”, and reduced discomfort after eating, alleviating instances of heartburn (Table 3, Quote 4.2.2.).

#### 3.4.3. Diagnosing

The process of diagnosing was highlighted as an important step in self-management. Without a diagnosis, participants reported questioning whether what they were experiencing was a result of menopause or another biological cause with similar symptoms such as old age or a low thyroid (Table 3, Quote 4.3.2.). Receiving a clear explanation of symptoms helped participants validate their experiences:

*“Menopause is so horrible, it’s very debilitating. Like between mood swings and diet, and the pure mental torture. You don’t know what’s coming around the corner next with bloody thing and no sleep, and then you have a GP and say, oh, that’s only the menopause.”*

(P252)

A diagnosis by a medical professional put their experiences into context and provided them with clarity as well as access to appropriate treatments, support for alleviating symptoms, and education on informed strategies for coping. Collectively, these strategies illustrate the diverse and proactive ways in which women navigated menopause, balancing medical interventions with self-directed lifestyle modifications.

## 4. Discussion

The purpose of this study was to determine whether sensory deterioration, namely in taste perception, happens during and after menopause, and to investigate how these changes affect menopausal women’s dietary habits and quality of life. The participants in this study experienced both diminished and heightened changes to sensory perception during their menopausal transition. Several women reported a decline in taste perception, with food tasting bland. This aligns with findings from a Japanese study where 77% of perimenopausal women experienced oral sensory complaints, including dysgeusia [52], and a German study linking reduced taste and smell sensitivity to poorer food-related quality of life [53]. Further evidence from Indian and Swedish studies suggests that postmenopausal women exhibit elevated taste recognition thresholds and a higher incidence of taste loss compared to premenopausal women or men, respectively, with hormonal influences being implicated [37,54]. Conversely, some women reported heightened smell sensitivity, finding odours overwhelming. Research indicates that oestradiol therapy and hormone therapy can improve olfactory test performance and odour detection thresholds in postmenopausal women [55], and oestrogen declines are suggested to influence olfactory function [56]. Regarding taste, some women experienced exaggerated sweetness or spice intolerance, alongside blunted taste. The NutriAct cohort highlighted marked inter-individual variability in taste sensitivity and its relationship to eating pleasure [53]. Experimental research demonstrates that oestrogen receptors are present in gustatory pathways and modulate receptor activity [57]. Taken together, these data suggest that fluctuating hormone levels during menopause may underlie heterogeneous taste responses.

The menopausal transition was associated with significant alterations in appetite and eating habits. Participants commonly highlighted increased snacking frequency, characterised by insatiable hunger and reduced satiety efficiency, leading to weaker fullness cues. Longitudinal evidence from the Canadian MONET cohort (*n* = 90) confirmed that eating frequency and hunger increase across the menopausal transition [58]. In addition, a French cohort study (*n* = 62,000) of women found that midlife women reported more frequent snacking and higher energy intake from discretionary foods postmenopause [59]. Similarly, the Australian Longitudinal Study on Women’s Health (*n* = 8000) reported increased snacking behaviours during menopause, particularly in women with vasomotor symptoms [60]. Oestrogen decline also amplified sugar cravings by altering reward circuitry, leading to increased consumption of sweet foods. Asarian and Geary demonstrated in animal models that ovarian hormone depletion increased sweet preference [61]. Human studies confirm similar trends: in a UK dietary recall study of 220 midlife women, postmenopausal women consumed significantly more added sugars than premenopausal controls [62]. Thus, oestrogen decline appears to amplify hedonic drive for sugar, but behavioural interventions can mitigate its impact. Furthermore, diminished taste sensitivity postmenopause is linked to a heightened preference for salt and increased sodium intake, which poses a cardiovascular risk. A U.S. NHANES analysis (*n* = 4000 women aged 40–65) showed that sodium intake increased significantly after menopause, often exceeding dietary recommendations [63]. While these cravings are influenced by hormonal changes, behavioural interventions can help mitigate increased intake of sugar and salty snacks. Food preferences shift, with a seeking of stronger flavours (e.g., chilli, coffee), the development of food aversions (e.g., to cucumber, red meat), and new preferences for previously disliked foods. These changes are supported by evidence from multiple cohort studies and research investigating hormonal influences and sensory perception during menopause [52,53]. This suggests that both physiological and behavioural adaptations may drive preference changes during menopause.

Changes in nutritional practices among menopausal women were shaped more by physical, emotional, and social wellbeing than by chemosensory changes alone. Focus group participants reported low motivation, reduced self-esteem, and diminished quality of life. Concerns around weight gain and body image were prominent, aligning with evidence from Silva et al. that declining oestrogen reduces lean body mass while increasing fat mass [10], and Lovejoy et al. observed increases in visceral abdominal fat postmenopause [64]. While these physiological changes are well-documented in altering self-image and confidence, participants’ accounts added an important qualitative dimension by highlighting the felt experience of weight gain as something sudden and uncontrollable. This loss of bodily autonomy was closely tied to diminished self-esteem and a disrupted sense of identity, with some women describing social withdrawal and reluctance to engage in previously enjoyed activities, including dining out with friends and family. Thus, the findings extend existing qualitative evidence by revealing how biological changes are experienced not merely as physical transformations but as challenges to self-image and social participation.

Despite challenges, women demonstrated agency through diverse coping strategies, including medical treatment, holistic therapies, and dietary changes. Receiving a diagnosis was described as pivotal, offering clarity, reducing uncertainty, and granting access to treatment. HRT was widely perceived as transformative, consistent with evidence of its effectiveness for vasomotor and psychological symptoms [65,66]. Additionally, participants experimented with holistic options such as sage, evening primrose oil, and magnesium, though often with caution given mixed evidence [67]. Anxiety was frequently reported, with some turning to prescription medication or hormonal devices such as the Mirena coil. Knowledge gaps were also evident: Aljumah et al. found women often felt uneducated and poorly supported by health practitioners, with some unaware they were experiencing menopause until after it had passed [68]. Focus group accounts echoed this, highlighting frustration when symptoms were dismissed—particularly by male practitioners—and demonstrating a lack of accessible resources. Other studies reinforce these findings: Lycke and Brorsson described inadequate healthcare support [69], while Hobson and Dennis highlighted workplace challenges for women in healthcare roles [70]. Together, these results underline the pressing need for professional, regulated, and competent services to support women through menopause, addressing both physical changes and wider social impacts.

### 4.1. Strengths and Limitations

Using Braun and Clarke’s thematic analysis provided a flexible and transparent framework for examining women’s lived experiences of menopause, allowing for rich insights into both individual and collective perspectives within the focus groups. This approach was particularly valuable for exploring sensitive issues around diet and health, as it enabled nuanced interpretation while maintaining methodological rigour. In addition, the results reflect the views of the women who participated and may not be generalisable to all women experiencing menopause. Focus group dynamics, such as more vocal participants influencing the discussion, may also have shaped the themes, and the qualitative nature of the analysis does not allow for quantification of findings or prevalence across wider populations.

A key limitation of this study is that demographic information (e.g., age, socioeconomic status, ethnicity, education, or occupation) of participants was not collected. This restricts the ability to contextualise findings, compare findings across subgroups, or explore how different demographic factors may shape women’s experiences of nutrition practices and chemosensory changes during menopause. Future studies should incorporate demographic profiling to strengthen the transferability and depth of insights.

### 4.2. Implications and Future Research

Future research should aim to deepen understanding of the mechanisms underlying chemosensory changes during menopause and their impact on dietary behaviours, with attention to how these experiences vary across different populations, life stages, and cultural contexts. Longitudinal and interdisciplinary studies would be particularly valuable in clarifying why these changes occur and how best to address them. In the interim, efforts should focus on developing practical resources, including support groups, enhanced clinical awareness, and improved nutritional guidance, to help women navigate the challenges of menopause and maintain quality of life beyond the transition. Education is also essential, not only for healthcare providers but also for family members and women across the life course, to foster greater awareness, reduce stigma, and ensure that appropriate support and interventions are accessible to all.

## 5. Conclusions

The study illustrates that menopause is characterised by a complex interaction of sensory, physiological, and emotional changes. Women’s eating habits and nutritional choices have been found to be influenced by altered taste perception and increased sensitivity to flavours and odours, which are frequently associated with weight fluctuations and other menopausal symptoms. These findings demonstrate significant individual variation in how women experience and cope with these changes. Future research should investigate the underlying mechanisms driving these changes and explore strategies to support women across different life stages.

## Figures and Tables

**Table 1:** Focus group guide questions and their importance for exploring the chemosensory changes during menopause.

Question	Research Importance
What are your initial thoughts when reading the case?	To gain insight on participants’ understanding and perspective.
Can you describe any similar cases from your own experiences? Tell us about what it was like and how you acted.	Encourages participants to discuss their personal experiences like that of the case study.
In which ways have you noticed changes in taste—more bland, bitter, sweet?	To gather knowledge more catered towards taste changes.
Has this affected your overall eating habits? e.g., more snacking.	Participants can discuss changes in their eating habits due to menopause.
Have these changes impacted your social life? e.g., not wanting to dine out.	To investigate if emotional and social status was changed due to chemosensory changes.
Have you found it hard to accept these changes? Have you applied any coping mechanisms? e.g., added more salt to food.	Analyses how participants cope with taste and smell changes.
Are there any foods you used to really enjoy and now find unappealing?	To determine whether taste changes occurred unbeknownst to participants.
Do you find you look for stronger tasting/smelling foods in order to enjoy what you are eating?	Investigates if menopause affects sensory perception and, if so, if it affects consumption of meals.

**Table 2:** Demographic characteristics of the female participants (*n* = 40).

Not Currently Taking HRT	28 (70)
Menopausal	12 (30)
Perimenopausal	1 (2.5)

**Table 3:** Themes, sub-themes, sample codes, and exemplar quotes identified in the thematic analysis.

Theme	Sub-Theme	No.	Sample Codes	Exemplar Quote
**1. Chemosensory Changes**	1.1. Diminished sensory perception	1.1.1	Sensory decline	“Well, taste for me is a big thing. I could be having breakfast, or any meal and it just tastes like I could be eating a bit of cardboard.” (A001)
		1.1.2		“I find that, (fast food restaurant name) and those kinds of things are very, I don’t know, they’re not as nice as they used to be.” (P791)
		1.1.3		“It’s not just bland, it’s incredibly sweet. It’s like really, really heightened. And that’s the only thing probably in my taste that I can really guess. Since the menopause is anything that is really, really sweet, I can still eat chocolate.” (B004)
		1.1.4		“I’m literally eating for the sake of eating because everything is just so bland. It doesn’t taste of anything.” (B004)
	1.2. Heightened sensory perception	1.2.1.	Smell sensitivity	“You know, if you cut an onion and if somebody throws it in the bin, I could smell it coming down the road” (B005)
		1.2.2		“Oh my God. My smell is heightened and it would trigger migraines. So roses, Onions. Particularly Taytos cheese and onion.” (B003)
		1.2.3		“No vanilla candles. I love candles, vanilla candles ugh rotten. No, I can’t smell them since menopause.” (B002)
		1.2.4		“My sense of smell has heightened without a shadow, without and say, like chicken. If chicken was best before the 3rd, I’d have to use it by the 1st, whereas if meat is in the fridge for two days, I’m like a wolfhound smelling it. And like my definitely my sense of smell has heightened and it does affect the food because the dogs have never been as well as well-fed here in the last three or four years.” (C005)
		1.2.5	Taste sensitivity	“Taste for me is a big thing, so I like. I could be having breakfast or any meal and it just tastes like I could be eating a bit of cardboard. Say toast for example. Even if I put on plenty of natural butter rather than spreads. You’d just be loving the dairy taste of it, but now it’s just I might as well be eating a leaf on top of my toast. I don’t get any major taste.” (A001)
		1.2.6		“There’s no real strong, enjoyable flavours for me.” (A001)
		1.2.7		“I haven’t noticed anything but taste. I cannot have a spicy Curry anymore. I cannot have anything spicy.”(B001)
		1.2.8		“I cannot have a spicy Curry anymore. I cannot have anything spicy.” (B001)
		1.2.9		“I would make mine and then they’d add more spice to it to it, like fajitas. You know, I would have it as normal and then somebody else would throw in extra, you know, just to spice it up a bit. And mine were probably too bland. But that’s how I like it. So it’s really, really just spicy stuff that I’ve noticed.” (B001)
		1.2.10		“Or chocolate, you know, like it, it actually feels way nicer and sweeter. And the cheese tastes nicer. You know, now maybe that’s because I’m not allowing myself as much, But by the same token, I think it definitely is heightened.” (B003)
		1.2.11		“Decrease taste. Yeah. But like I just, spice it out of it. But there was a little there was a decrease. Things were tasting bland. More salt more.” (C005)
	1.3. Appetite and Eating Habits (snacking)	1.3.1	Increased snacking	“I sit down and I’m in and out of the ***** cupboard for the rest of the evening.” (C002)
		1.3.2		“I’m kind of a grazer every few hours… Probably more so in the last few years.” (P406)
		1.3.3		“More snacking… Hungry. Hungry, hungry. The whole time.” (A005)
		1.3.4	Appetite changes	“My appetite. I couldn’t quench it. I was hungry all the time, even eating load of protein which I eat lot of protein. I’m still hungry. I wasn’t satisfied. But with the last few months now I’m grand. I can go 5 h without eating and not be hungry.” (P864)
		1.3.5		“Still, I’m so hungry, and if I’m eating with people, I’m embarrassed to sort of go. I’m still hungry, so I might go home and have something else. But, like, my appetite is just ridiculous”. (P110)
		1.3.6		“I eat a lot more. I can’t stop eating and I could eat what somebody else hasn’t finished on their plate. I just, I cannot be you know, full enough, most of the time”. (P110)
		1.3.7		“But I am eating an awful lot more. I’m eating more than my partner, like he doesn’t finish his dinner, and I’ve finished mine. And I’m like OK, give us it here.” (P110)
		1.3.8	Increase in sugar intake	“I love sweets. And definitely since the menopause I would be sweet mad. Sweet, sweet mad.” (P101)
		1.3.9		“For me, now one time I would never invest biscuits or cakes too much. It wouldn’t that they wouldn’t have bothered me. But over the last number of years, definitely, you know, would definitely buy a packet of biscuits.” (A001)
		1.3.10		“One time I used to make a big sandwich of lettuce and maybe a bag of crisps in it and all that kind of stuff, I’d have no interest. Not now, but I might think of a biscuit more so now than that.” (A001)
		1.3.11		“I do have cravings for chocolate, So I think maybe that could be the sweet thing. It’s crazy. Every evening, I come home after dinner. It’s like, what’s in the cupboard?” (B001)
		1.3.12	Increase in salt intake	“I love salt. I love Aromat. They’re the two things that I can’t live without on food. I just need the flavour to be honest…And people do comment on it, that’s an awful lot of salt, you know but I’m like really? I can’t taste it.” (P110)
		1.3.13		“Yeah, love salt. I’d put salt on ice cream.” (P252)
		1.3.14		“At one time I never used salt and anything in cooking or anything at all, but now I would find that I’d need salt or need to flavour things a bit more” (P614)
		1.3.15		“I never even knew what Aromat was until a few years ago. And yeah, sometimes I’ve just mixed it up a bit, I think I won’t have salt, I’ll have Aromat. But yeah, I just need the flavour to be honest. And people do comment on it. Jeez, that’s an awful lot of salt, but I’m like, really, I can’t taste it. So yeah, I’ve always liked salt, I’ve really just gone a lot more since in the last couple of years.” (P110)
		1.3.16		“I don’t know if it’s menopause. I just crave cheese all the time anyway. I just like cheese is my thing, and that’s probably maybe craving salt or something as well. That kind of umami flavour.” (B005)
		1.3.17		“I actually I use it celery salt because you know the way they say keep down your intake of salt. But Jesus I’d be. I’d be picking up the salt every 3 min. Do you know? And I’d eat the top layer and then more salt and more, you know? And like, yeah, things are a bit blander.” (C005)
	1.4. Food Preference (cravings/aversions/flavours)	1.4.1	Seeking stronger flavours	“I want some chilli on everything.” (C002)
		1.4.2		“I’ve started having that extra spoon of coffee into my coffee.” (P864)
		1.4.3		“I’ve developed my palate a lot more, I would have been very, very fussy at one stage when I was younger, but definitely like to taste different foods and more spice. Well, not spicy , but you know, I would steer away a little bit from the stews, the bacon and cabbage and the roast chicken and veering over towards the other direction towards the Indian. Just for the that sense of having something different. I think yeah, two years ago, I would have been a bit resistant to even trying things like that.” (P817)
		1.4.4		“Yes, spicier if I couldn’t get away with it yet. Yeah. Curries. Yeah. Yeah. Whereas before I used to hate, anything with chilli in it. Forget it. Now, just give it to me. I’m fine.” (B004)
		1.4.5		“I just needed a stronger flavour. Just need a kind of something spicy.” (C002)
		1.4.6	Avoidance of certain foods	“And I used to love cucumber. I can’t. I just find it disgusting. And now it’s everything about it, just the smell, the taste, it just makes me heave.” (C002)
		1.4.7		“When I’m putting in the chilli powder or the paprika or anything (into chilli con carne), I just it just turns me off, you know that kind of way. So I don’t know if that makes sense.”(A006)
		1.4.8		“Even spices like paprika or spices like that that I would have always thought were OK. They just give me the gawk.” (A006)
		1.4.9		“I used to eat a steak every time, I loved it. Just couldn’t look at it now, I have no interest in the world.” (P651)
		1.4.10	Change in preferences	“I actually now eat gherkins. I never liked gherkins. Well, I do eat gherkins, but these are the vinegary taste. That’s something” (P864)
		1.4.11		“I like ginger now, so I can’t eat milk chocolate anymore. It’s very sickly when I eat that dark chocolate I’ve eaten dark chocolate for a little while now anyways, and probably just the palette’s probably changed, but I eat like 85 even 90% dark chocolate, but I started adding the frozen ginger.” (P864)
		1.4.12		“The thing that actually surprises me is I’m actually able to eat bananas now, which I never was. I used to hate the taste of them and the texture of them. I used to hate and they used to give me headaches, but now no problem, whatever that’s about.” (B004)
**2. Behavioural and Emotional Consequences**	2.1. Weight gain and Body Image	2.1.1	Unconscious weight gain	“I never in my whole life had to go diet at all. I was always thin and could eat all around me. And you know, it’s just depressing to see what happens to your body when you get old and you can’t control it?” (P110)
		2.1.2		“I just found that, yeah, the weight has heaped on, the minute I hit 50, I felt like I gained about an extra stone and a half like in a week.” (C003)
		2.1.3		“I find is I have loaded on, I’ve 3 stone put on and I just. Can’t get it off between everything, between not being able to exercise, it just seems to be loading itself on. (P261)”
		2.1.4	Weight gain during menopause	“You definitely put on weight way easier, I watched what I ate.” (P922)
		2.1.5		“I had put up weight kind of coming into menopause I would say.” (C004)
		2.1.6		“My weight loss journey was so, so different to what the girls are feeling now because I put on so much weight we had a couple of things going on and I just didn’t care about anything and just the weight just piled on or whatever. I didn’t want to go anywhere.” (C004)
		2.1.7	Changing body shape	“But the menopause weight is different. I’m a barrel now. It’s a barrel. Like it’s it. Just it kind of lands on you and doesn’t want to go.” (C005)
		2.1.8		“I haven’t had much change in weight, but certainly body shape has changed and that abdominal or that tummy fat and maybe thighs, there’s certainly a bit more condition on there without a change in the scales.” (P614)
		2.1.9		“I’ve never had weight on my abdomen but I over the last year, so I’ve gone up a stone and a half and also now I’m hoping to get that down again. But it’s our body will actually make you eat more because it wants more fat basically and that’s why my appetite went up because I never had weight not for a long, long time where now I’m up a stone and 1/2.” (P864)
		2.1.10	Influence of cravings	“When you were putting on the weight, the craving was always there for the sugar, the sugary stuff you know, like the chocolate.” (P311)
		2.1.11		“I eat a lot more. I can’t stop eating and I could eat what somebody else hasn’t finished on their plate.” (P110)
	2.2. Support Network	2.3.1	Family	“Is there a difference where you come in the family? If you have a lot of sisters or older members and the families…some of us may not have had anyone to talk to.” (P651)
		2.3.2		“Mammy had gone through it young and it looked like there was a more chance of me going through it earlier as well, which I think is like you don’t like a lot of people getting married older and things and having kids older now.” (P791)
		2.3.3		“…family history, you find if peoples’ mother goes through the menopause early… But she said her mother had gone through. It’s quite young as well. So there’s something to do with history, family history as well, you know.” (A004)
		2.3.4	Forums/focus groups/courses/specialist	“It would help, particularly younger people…if I had my time over I would definitely arm myself with more information and there’s support groups and all of that they like even chatting about it tonight. If those people can go on to different forum and you know get help through groups… Definitely would make a difference, one would say look, I tried this and I tried that and this worked.” (P252)
		2.3.5		“Apart in my doctor’s office I’ve never spoken openly about the subject to anyone unless on a form to see.” (P651)
		2.3.6		“I’ve done a lot of research into the menopause and I’m just about to do a course. The sad thing is, a lot of women feel they never have menopause symptoms, but 100% of women go through menopause. You have senses from head to toe, so it might be just a waking up during the night. You might not realise like new onset of asthma, frozen shoulder joint pain, hip pains cracking knees. All those can be a sign of low oestrogen.” (P864)
		2.3.7		“I went to a menopause specialist. In the women, Well, Women’s Centre, because I really I was struggling” (C005)
**3. Interacting Influences**	3.1. Physical symptoms	3.1.1	Sleep disturbances	“I would have awful leg cramps. I would be in and out of the bed every night, not sweating.” (P101)
		3.1.2		“I think, across the board is insomnia. Broken sleep. That’s every night, every night without a shadow of a doubt. If I had a full night’s sleep, I’d be celebrating at the morning.” (P252)
		3.1.3	Joint pain and stiffness	“I had joint pains. I’d get up in the morning, it was like your hips had to realign, and the muscles had to get into place.” (P922)
		3.1.4	Hot flushes and sweats	“Definitely the hot flushes were a big issue for me and even during the day, I mean my clothes would be stuck to me, but I’m still at this stage where I get the night sweats. I’m so used to it now.” (P406)
		3.1.5		“I would have had a lot of night sweats as well and leg cramps”. (P341)
		3.1.6		“I don’t think I ever like broke out in the sweats, but it was just my face that I was just, you know, just that. It was very embarrassing. I think that’s like the only word. Like just you’re just in the middle of a conversation and then suddenly and you just want to leave the room. You just wanted to hide. It was awful.” (C002)
		3.1.7	Discomfort after eating	“I eat something, and then I have heartburn I’d be regurgitating after it. Banoffee pie. I can’t eat it anymore because of the banana. I’d be regorging all the time or feeling horrible and nauseous” (A001)
	3.2. Emotional wellbeing	3.2.1	Lack of motivation	“My taste is fine. It’s the get up and go for me’”(C003)
		3.2.2		“To me, everything’s really bland now. Yeah. So I wouldn’t really. I wouldn’t be craving anything anymore.” (B004)
		3.2.3		“It wouldn’t really matter to me if I was having a nice dinner or if I was having just a banana sandwich. They all kind of just felt blah” (C004)
		3.2.4	Loss of identity	“I just wish I could go back to the old me. And blame the menopause for everything” (COO3)
		3.2.5		“For me, it’s the tiredness is the biggest thing and the weight…it gets me down and I don’t want to go out, I don’t want to be in public. I literally get up. I go to work, I come home and the door is closed. I wish I could get my mojo back and be the fun again.” (C003)
		3.2.6	Brain fog	“I’m even surprised I can remember the name of the thing that I’m eating with the brain fog sometimes. You know, you feel like saying to the kids, what’s the name of this thing?” (B005)
	3.3. Social wellbeing	3.3.1	Embarrassment over increased appetite	“I’m so hungry, and if I’m eating with people, I’m embarrassed to say ‘I’m still hungry’, so I might go home and have something else. My appetite is just ridiculous.” (P110)
		3.3.2	Lack of desire to dine out	“I don’t have a mad desire for it. I’m not as interested as I used to be.” (A001)
		3.3.3		“I was having a bit of a panic attack because I just, you know, just I just felt so fat like a little barrel everything I put on was just stuck to me. And I would have loved to have cancelled that night rather than go out. I just. Yeah, I wasn’t. I mature that night and I just, I didn’t want to be out. I didn’t want to be out in public. I didn’t feel a bit comfortable.” (C002)
**4. Strategies for Wellbeing**	4.1. Treatment—medical and holistic	4.1.1	Physician prescribed—hormone replacement therapy (HRT)	“Wouldn’t be without it. God, no, no.” (P198)
		4.1.2		“Ringing in the ears. That was one of the symptoms I got…but that the ringing is gone now since I started HRT.” (P864)
		4.1.3		“Before I went on HRT like I was very emotional. Like you could literally just pay me a compliment and I could burst into tears. Or you could say hello. And I’d want to punch you in the face…I’d cry at the drop of a hat. I’d want to throw things and scream. It was horrendous. Like I just thought I was going mental” (P110)
		4.1.4	Physician prescribed—prescription medication	“I’ve had been on medication. I’m just coming off it now. I can’t even pronounce it. But it’s like an anxiety tablet, but it helps with the hot flushes, which it did.” (A005)
		4.1.5		“The Mirena (coil) contains progesterone, which is one of the hormones that we lose and that’s why it’s so good for peri and menopausal symptoms because it’s producing the tiniest little bit of progesterone into your body. So stay on it as long as you can, as the doctor said, because it could be just keeping up the progesterone level, which is superb. And it is recommended a lot.” (P922)
		4.1.6	Holistic therapy	“I got a magnet. That was very good. It was just a magnet. It’s actually pushed inside you just under your belly button, but it stopped the sweating with me.” (P791)
		4.1.7		“The only thing I had been taking is evening primrose. And I found it very good.” (P212)
		4.1.8		“I found organic sage very good for flushes and a friend of mine who’s a nurse. She suggested it to me. Organic sage comes in a capsule and you get it in the health food shop. And I just found it’s brilliant for calming down the flushes.” (P261)
		4.1.9	Magnesium	“I used to get kind of leg cramps sometimes and while just when you mentioned there. I don’t think I’ve got them since I was on the magnesium” (A006)
		4.1.10		“Magnesium is good. I’ve got the liquid now of the oil that you rub it on to your you know. So if you sore legs.” (A005)
		4.1.11		“You have to be careful that you need to be led by a herbalist or something, because if it’s not that problem, you’ll create a new problem by taking it (magnesium)” (P252)
	4.2. Food modification and conscious eating	4.2.1	Modifying food behaviour	“I’d be avoiding the spicy foods. I would be like ‘just give me spaghetti bolognese’. No, I stay away from the curries. Smaller portions that would be my thing” (B001)
		4.2.2	Dietary improvement	“I think from a point of view of eating healthier, I would definitely try and eat fresher and healthier… I suppose I’m consciously thinking about not having a high sugar spike…. So if I’m eating less sugar, but I still like, I enjoy everything, but I’d be more conscious about health.” (B003)
		4.2.3	Conscious consumption of foods	“I would try and eat less. I taste it more, I’d eat less, but I’d taste it more” (B003)
		4.2.4		“In the evenings, kind of not leaving it too late, but just maybe a bit more aware of how much I’m eating than trying to be a bit more disciplined” (B005)
		4.2.5		“But yeah, but smaller portions. That’ll be my thing.” (B001)
		4.2.6		“I just have to be bit careful. Like there’s certain foods anyway, like I’ve no portion control over certain things. You know, I just like pasta” (B005)
	4.3 Diagnosing	4.3.1	Confusing factors associated with diagnosing	“I don’t know is it menopause. Is it low thyroid? Is it old age?” (P413)
		4.3.2		“But some of the symptoms that I find from my own experience, some of the symptoms of menopause and the symptoms of low thyroid overlap. Some of the symptoms are very much the same. You can have hot flushes and brain fog. You can have the pains, you can have all of them things. So I never, I don’t know what’s wrong with me really.” (P413)
		4.3.3		“I ticked all of her symptoms (referring to the case study), I have them all and probably more. So, yeah. It’s hard to know how much of it you can put down to ageing and how much of it is menopause, but either which way, I have nearly everything she has and more.” (C001)
		4.3.4	Support with symptoms	“I have heard that allergies are very common in some people during menopause. It’s a histamine issue and it can be looked into. You possibly have too much histamine in the system.” (P198)
		4.3.5		“Because we’ll have lost our hormones and you can’t replace them unless you take HRT, so that’s some of the new teaching.” (P922)

## Data Availability

The data that support the findings in this article are included within the article.

## Appendix A

PATIENT CASE STUDY (to be read out during Focus Group)

Jane

Jane is 50 years old and is currently going through menopause. Jane is almost fully gone through menopause and has experienced various symptoms since beginning.

Jane has recently complained about losing her taste for coffee. Before this Jane stated she would have at least 2 cups of coffee a day, “if not more”, and now doesn’t even want half a cup of coffee. Jane has also found her tolerance for spices has deteriorated, she used to have a high tolerance for spices but now finds a mild curry spicy, she has found her tastebuds have heightened and struggles to eat stronger foods.

Jane has recently started to eat healthier and exercise more regularly, losing almost 3 stone of weight. She found when she was heavier that symptoms were more enhanced, such as having pains in her legs and joints.

Asides from taste changes, Jane has noticed having hot flushes but less frequently since losing weight, these hot flushes didn’t cause sweating. As well as this she finds it wakes her in the middle of the night at the same time each night. Jane feels her eyesight has also gotten worse since starting menopause but hasn’t got an eye-test since to confirm. She hasn’t noticed any changes in her hearing.
